# LPS-induced mitochondrial DNA synthesis and release facilitate RAD50-dependent acute lung injury

**DOI:** 10.1038/s41392-021-00494-7

**Published:** 2021-03-03

**Authors:** Xueqin Zhan, Rui Cui, Xinwei Geng, Jiaqian Li, Yunlian Zhou, Lulu He, Chao Cao, Chao Zhang, Zhimin Chen, Songmin Ying

**Affiliations:** 1 Department of Pulmonology, Children’s Hospital, Zhejiang University School of Medicine, National Clinical Research Center for Child Health, National Children’s Regional Medical Center, Hangzhou, Zhejiang, China; 2 International Institutes of Medicine, the Fourth Affiliated Hospital of Zhejiang University School of Medicine, Yiwu, China; 3 Department of Pharmacology and Department of Respiratory and Critical Care Medicine of the Second Affiliated Hospital, Zhejiang University School of Medicine, Key Laboratory of Respiratory Disease of Zhejiang Province, Hangzhou, Zhejiang, China

**Keywords:** Inflammation, Respiratory tract diseases

**Dear Editor**,

ATP-binding cassette (ABC)-ATPase (RAD50), together with meiotic recombination 11 homolog 1 (MRE11) subunits, to form MRE11–RAD50 complex, plays important roles in recognition of double-stranded DNA (dsDNA) and initiation of consequent inflammatory cascade^1^. Acute lung injury (ALI) and acute respiratory destress syndrome (ARDS) are systemic uncontrolled inflammation and life-threatening. However, the function of the DNA sensor in ALI/ARDS remains poorly defined. Here we investigated functions of RAD50 using mouse primary macrophages and conditionally RAD50 knockout mice in vitro and in a lipopolysaccharide (LPS)-induced lung injury model.

Emerging evidence suggests that mitochondrial DNA (mtDNA), the only form of extranuclear dsDNA in eukaryotic cells, is a major activator of inflammation when leaked out from stressed mitochondria^2^. Increased mtDNA level in plasma has been reported to be associated with incident ARDS in trauma and sepsis patients^3^. However, the mechanism of mtDNA-induced inflammation in ALI/ARDS still needs to be elucidated. Macrophages are the first responders of pulmonary innate immune system. Here, we observed that primary macrophages exhibited a higher extranuclear dsDNA level under LPS stimulation (Fig. 1a). LPS-TLR4 engagement has been reported to contribute to MyD88/TRIF-dependent signaling to induce mitochondrial deoxyribonucleotide kinase CMPK2 and dNTP hydrolase SAMHD1 expression in bone marrow-derived macrophages^4^. Then we investigated whether these dsDNA originated from the nucleus or mitochondria. Primary macrophages were treated with LPS in the presence of 5-ethynyl-2′-deoxyuridine (EdU) to measure mtDNA replication, and the result showed that LPS resulted in a rapid and remarkable increase in extranuclear DNA synthesis (Fig. 1b). Besides in mitochondira, these newly synthesized DNA also appeared in the cytosol (Fig. 1b). Moreover, LPS induced an increase of mitochondrial reactive oxygen species (ROS) level (supplementary Fig. S1). Additionally, we examined nuclear DNA damage markers γH2AX and RPA at the same time, and found that nuclear DNA fragmentation did not occur in the primary macrophages with LPS stimulation (supplementary Fig. S2). These findings further substantiated LPS induced nascent mtDNA synthesis, promoted its release into the cytoplasm, and triggered an increase in extranuclear dsDNA.

**Fig. 1:**
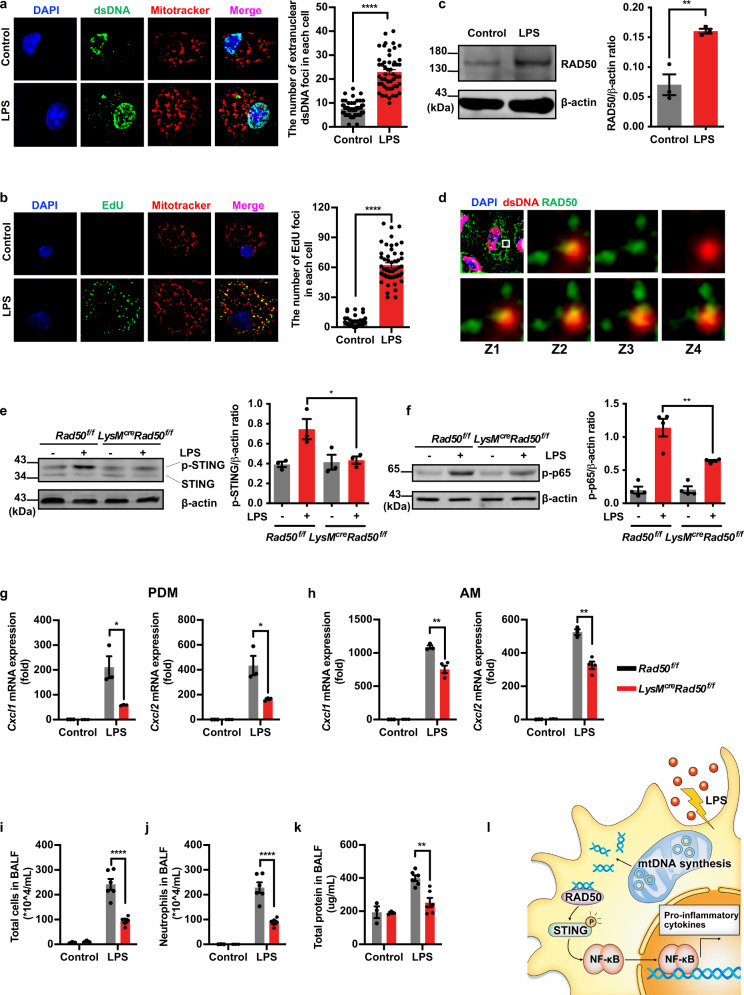
*Rad50* deficiency alleviated LPS-induced inflammation in macrophages both in vitro and in vivo. **a**–**h** Primary macrophages from *Rad50*^*flox/flox*^ or *LysM*^*cre*^*Rad50*^*flox/flox*^ mice were administrated with LPS for 6 h. **a** Representative structured illumination microscopy images of macrophages of extranuclear dsDNA and the statistical analysis of the number of extranuclear dsDNA foci in each macrophage. **b** Representative structured illumination microscopy images of macrophages stained with EdU (newly synthesized DNA) and mitotracker (mitochondria) were shown and the number of EdU foci in each macrophage was counted. (50 cells at least per group; N-SIM, original magnification, ×400). **c** The expression of RAD50 was determined by western blot. **d** Representative structured illumination microscopy images of primary macrophages, which were stimulated with LPS and stained with dsDNA and RAD50 antibodies, and the co-localized foci for dsDNA and RAD50 in various z-layers (z1–z4) in remaining images. **e**, **f** Western blot was performed to examine the activation of STING and the expression of p-p65. **g**, **h** Relative levels of *Cxcl1* and *Cxcl2* of peritoneal-derived macrophages (PDMs) and alveolar macrophages (AMs) were determined using quantitative PCR. **i**–**k** *Rad50*^*flox/flox*^ and *LysM*^*cre*^*Rad50*^*flox/flox*^ mice (*n* = 3–6) were instilled intratracheally with LPS (1.25 mg kg^−1^) or the equivalent volume of PBS as control for 12 h. In the bronchoalveolar lavage fluid (BALF), **i** the total number of inflammatory cells was quantified, **j** the number of neutrophils was calculated, **k** BCA assay was used for measuring protein concentration in BALF. **l** Schematic overview of the function of RAD50 in LPS-induced ALI. All experiments were repeated at least three times. Data are presented as means ± SEMs. *p < 0.05, **p < 0.01, ***p < 0.001, ****p < 0.0001

In the cytoplasm, there are multiple DNA sensors, such as IFI16, MRE11/RAD50/NBS1 complex, to maintain a dsDNA-free environment^1^. We examined the expression of DNA sensor RAD50 in LPS-stimulated macrophages. As shown in Fig. 1c and d, LPS stimulation caused increased RAD50 expression, and the cytoplasmic delivery of dsDNA resulted in the recruitment of RAD50, with the formation of distinct dsDNA-RAD50 foci in the cytoplasm.

MRE11 was previously reported to be located in the mitochondria, and MRE11 deficiency causes mtDNA release into the cytosol^5^, but the function of RAD50 in the process of cytoplasmic mtDNA leakage in macrophages remains unrevealed. We crossed *LysM-cre* and *Rad50*^*flox/flox*^ mice to generate *LysM*^*cre*^*Rad50*^*flox/flox*^ mice that exhibit deficient RAD50 in myeloid cells, including macrophages. The level of RAD50 was significantly decreased in macrophages from *LysM*^*cre*^*Rad50*^*flox/flox*^ mice (Supplementary Fig. S3). Primary macrophages from control mice (*Rad50*^*flox/flox*^) and *LysM*^*cre*^*Rad50*^*flox/flox*^ mice were stimulated with LPS in the presence or absence of the EdU. The results showed that LPS-induced extranuclear newly-synthesized DNA or extranuclear dsDNA was unaffected by RAD50 ablation (Supplementary Fig. S4a, b). Furthermore, RAD50 exhibited no significant protection on mitochondria injury, which included mitochondrial ROS level and morphological change (Supplementary Fig. S4c, d).

The detection of damaged and mislocalized DNA in the cytoplasm leads to activation of stimulator of interferon genes (STING) and downstream IRF3 and nuclear factor kappa-B (NF-κB) -mediated production of type I interferon and other pro-inflammatory cytokines^1^. We then investigated whether RAD50 mediates the activation of STING signaling pathway under LPS treatment. As shown in Fig. 1e, STING activation was increased, and those with *Rad50* deficiency exhibited diminished activation of STING. Additionally, we observed that depletion of *Rad50* reduced the phosphorylation of NF-κB p65 (Fig. 1f) and also decreased LPS-induced expression of cytokines *Cxcl1*/*Cxcl2*, which are regulated by the NF-κB signaling pathway (Fig. 1g, h). We also performed additional experiments about two other proteins of MRE11/RAD50/NBS1 complex and found that MRE11 was also potentially required for LPS-induced macrophage inflammation, whereas NBS1 was dispensable (Supplementary Fig. S5).

RAD50-dependent LPS-induced macrophage inflammatory response in vitro and relatively high *Rad50* expression in macrophages (Supplementary Fig. S6) implicate the function of RAD50 in acute lung inflammation. After substantiating the LysM-cre gene editing exerted no effects (supplementary Fig. S7), *LysM*^*cre*^*Rad50*^*flox/flox*^ and *Rad50*^*flox/flox*^ mice were used to establish an ALI model. *Rad50* myeloid-specific knockout mice (*LysM*^*cre*^*Rad50*^*flox/flox*^ mice) exhibited a remarkable decrease of inflammatory cells in bronchoalveolar lavage fluid (BALF) compared to *Rad50*^*flox/flox*^ mice (Fig. 1i, j). Similar protective effect on neutrophil recruitment was observed in an acute peritonitis model (Supplementary Fig. S8). We further found that the protective effects of *Rad50* deficiency on inflammation in ALI were reflected as reduced concentration of total protein in BALF (Fig. 1k) and attenuated airway pathology (Supplementary Fig. S9a).

Increased expression of inflammatory cytokines is important features of ALI. The consequent inflammatory cascade triggered by these cytokines has a great effect on the development of ALI. Supplementary Fig. S9b and S9c showed a dramatic reduction in the pro-inflammatory mediators *Cxcl1* and *Cxcl2* in the lungs by conditionally RAD50 deletion in myeloid cells in vivo. Additionally, *LysM*^*cre*^*Rad50*^*flox/flox*^ mice exhibited decreased phosphorylated p65 (p-p65) expression and STING activation (Supplementary Fig. S9d). These data indicated that RAD50 facilitated LPS-induced inflammation in vivo.

Taken together, we determined a previously unknown function of DNA sensor RAD50 in LPS-facilitated inflammatory response in innate immunity. We found that LPS induced mtDNA synthesis and release, and caused increased cytosolic dsDNA, which was recognized by RAD50 in macrophages. This important process consequently contributed to the activation of STING and NF-κB signaling pathway, and promoted pro-inflammatory cytokines release. The attenuation of acute lung inflammation mediated by *Rad50* deficiency suggested that DNA sensors might be promising therapeutic drug targets for the inhibition of systemic uncontrolled inflammatory response in ALI/ARDS.

## Supplementary information

 supplemental material

## Data Availability

The data during the current study are available from the corresponding author on a reasonable request.
