# Supplementary material for: LPS-induced mitochondrial DNA synthesis and release facilitate RAD50-dependent acute lung injury
Source: Signal Transduct Target Ther. 2021 Mar 3;6:103. doi: 10.1038/s41392-021-00494-7 (PMC7925566; doi:10.1038/s41392-021-00494-7)
Supplement: Supplementary file 1 — supplemental material [file 41392_2021_494_MOESM1_ESM.pdf]

Supplementary Materials for

LPS-induced mitochondrial DNA synthesis and release facilitate RAD50-dependent acute lung injury

Xueqin Zhan[#1,2], Rui Cui[#2,3], Xinwei Geng[#2,3], Jiaqian Li[2,3], Yunlian Zhou[1], Lulu He[3], Chao Cao[3], Chao Zhang*[3], Zhimin Chen*[1] and Songmin Ying*[2,3]

[1]Department of Pulmonology, Children's Hospital, Zhejiang University School of Medicine, National Clinical Research Center for Child Health, National Children's Regional Medical Center, Hangzhou, Zhejiang 310052, China.
[2]International Institutes of Medicine, the Fourth Affiliated Hospital of Zhejiang University School of Medicine, Yiwu 322000, China.
[3]Department of Pharmacology and Department of Respiratory and Critical Care Medicine of the Second Affiliated Hospital, Zhejiang University School of Medicine, Key Laboratory of Respiratory Disease of Zhejiang Province, Hangzhou, Zhejiang 310009, China.

*Correspondence: Songmin Ying (yings@zju.edu.cn), Zhimin Chen (chenzhimin6@163.com) or Chao Zhang (zhangchao001@zju.edu.cn)
[#]These authors contributed equally: Xueqin Zhan, Rui Cui, Xinwei Geng.

This PDF file includes:

Materials and Methods
Figures. S1 to S9
References

Materials and Methods

Mice

The *LysM-cre* mice (C57BL/6 background)[1] were provided by Dr. Gen-Sheng Fen (University of California at San Diego, CA, USA). *Rad50*$^{flox/flox}$ mice[2] (mixed C57BL/6 and 129Sv background) were purchased from Jackson Laboratory. *LysM-cre* mice were mated with *Rad50*$^{flox/flox}$ mice to generate myeloid cell-specific *Rad50* conditional knockout mice (*LysM*^{cre}*Rad50*$^{flox/flox}$). All mice were housed in a specific pathogen-free animal facility at Zhejiang University Laboratory Animal Center. All animal procedures were approved by the Ethics Committee for Animal Studies at the Zhejiang University. The primers used for genotyping are described below.

Rad50$^{flox/flox}$
Forward primers: CATGATCCCAAGGTAATGGTG
Reverse primers: TGCATGACACAAGGTCAGAGA
LysM-cre
Wild type: TTACAGTCGGCCAGGCTGAC
Mutant: CCCAGAAATGCCAGATTACG
Common: CTTGGGCTGCCAGAATTTCTC

Reagents

Ultrapure LPS (*Escherichia coli O555:B5*) was from Sigma-Aldrich (Merck KGaA, Darmstadt, Germany). Click-iT EDU Alexa Fluor™488 imaging Kit was from Invitrogen (Thermo Fisher Scientific, MA, USA). Antibodies against dsDNA (#ab27156, Abcam, Cambridge, MA, USA), actin B (#sc-47778, Santa Cruz Biotechnology, Texas, USA), RAD50 (#ab89, #ab124682, Abcam), γH2AX (#05-636, EMD Millipore, Birrika, Massachusetts, USA), RPA (#ab2175, Abcam), phospho-NF-κB p65 (Ser536) (#3033, Cell Signaling Technology, Danvers, MA, USA), and STING (#13647, Cell Signaling Technology) were used. Antibodies for immunofluorescence were diluted at 1:1000 in 5% BSA (#B2064, Sigma-Aldrich). Antibodies for western blot were diluted at 1:1000 in Western Primary antibody dilution (#P0023AS, Beyotime, China). All primers were synthesized by TSINGKE (Beijing, China). ELISA kits for mouse CXCL2 (#70-EK2142/2-96) were purchased from Multiscience Biotech (Hangzhou, China).

Cell culture

Peritoneal-derived macrophages (PDMs) were extracted from *Rad50* myeloid cell-specific conditional knockout mice and age- and sex-matched wild-type controls. Mice were injected 3% thioglycollate (EMD Millipore) intraperitoneally for 2 days, and then they were sacrificed on the day 4. PDMs were collected through peritoneal lavage and plated at a density of 1×10^6 cells·mL^{-1} in almost experiments. Alveolar macrophages (AMs) were obtained from the whole lung through alveolar lavage. All cells were cultured in RPMI 1640 (#C11875500BT, Gibco, Thermo Fisher Scientific) with 10% fetal bovine serum (#10270106, Gibco, Thermo Fisher Scientific) and 1% penicillin-streptomycin (#C0222, Beyotime) in 5% CO_2 at 37°C. After LPS (10 μg·mL^{-1}) stimulation for 6 hours, cells were subjected to further analysis.

siRNA transfection

Primary macrophages (6×10^5 cells·mL^{-1}) were seeded in 12-well plates for 2h, then siRNA was transfected into the cells using the a siRNA transfection reagent (Invigentech, USA) for 24h following the manufacturer's instructions. siRNA sequences are described below.

si*Mre11*:
Forward: 5′-GAGGCUUUGAACCUUUCAATT-3′,
Reverse: 5′-UUGAAAGGUUCAAAGCCUCTT-3′;
si*Nbs1*:
Forward: 5′-GCUGUCUUAACAGUAAACUTT-3′,
Reverse: 5′- AGUUUACUGUUAAGACAGCTT-3′;

RNA isolation and qRT-PCR

Total RNA of lung tissues and cells was isolated using TRIzol reagent (Takara Biotechnology, Kusatsu, Japan) or RNeasy kit (Qiagen, Germany) according to manufacturer's instructions and then reverse transcription was used to generate cDNA using a reverse transcription kit (Takara Biotechnology). Quantitative real-time PCR was performed using SYBR Green Master Mix (Takara Biotechnology) on the StepOnePlus PCR system (Applied Biosystems, Foster City, CA, USA). A ΔC_t method was performed to quantify mRNA levels. Samples were assayed in triplicate. Primer sequences are described below.

Actin B:
Forward: 5′-GTCCACCGTGTATGCCTTCT-3′,
Reverse: 5′-CTCCTGGTGTCCGAACTGAT-3′;
Cxcl1:
Forward: 5'-CTGGGATTCACCTCAAGAACATC-3',
Reverse: 5'-CAGGGTCAAGGCAAGCCTC-3';
Cxcl2:
Forward: 5'-TGTCCCTCAACGGAAGAACC-3',
Reverse: 5'-CTCAGACAGCGAGGCACATC-3';

Western blot

Protein from cells or lung tissues was extracted using RIPA lysis buffer (#P0013B, Beyotime), which contained protease inhibitors (#04693116001, Roche Diagnostics GmbH, Germany) and phosphatase inhibitors (#04906845001, Roche Diagnostics GmbH). The protein concentration was measured using a Pierce™ bicinchoninic acid (BCA) kit (#23225, Thermo Fisher Scientific). After quantification and denaturation, equal amounts of protein were run on sodium salt-polyacrylamide gel electrophoresis and then transferred onto a 0.2 μm PVDF membrane (#ISEQ00010, Millipore) that was incubated with the relevant antibodies. β-actin served as a protein internal control. The gray value assay of the sample was assessed using Image J software.

Immunofluorescence staining

Primary macrophages on coverslips with the pre-designed treatment were fixed in 4% paraformaldehyde for 15 minutes at room temperature, and permeabilized with 0.5% Triton-X-100 (#V900502, Sigma-Aldrich) for 20 minutes. After blocking with 5% bovine serum albumin (BSA) (#B2064, Sigma-Aldrich) for 40 minutes, the cells were incubated with the corresponding primary antibodies for 10 hours at 4°C. They were then incubated with Alexa Fluor®555 or Fluor®488-conjugated secondary antibodies against rabbit or mouse immunoglobulin G heavy and light chain (#A21424, Invitrogen) for 1 hour. To measure mtDNA replication in primary macrophages, in the presence of 10 μM EdU, the cells were treated with LPS (10 $\mu g \cdot mL^{-1}$) for 6 hours and then were incubated with mitotracker (500uM) (YEASEN Biotech, China) for 30 minutes. After cell fixation, permeabilization and blocking, EdU staining was performed according

to the manufacturer's protocol using a Click-iT™ EdU Alexa Fluor™ 488 Imaging Kit (Invitrogen). The nucleus was counterstained with DAPI for 10 minutes. Fluorescent images were captured with an automated Nikon Elipse Ni microscope or SIM microscope with Nikons-Eliments software (Nikon instruments).

Acute lung injury model

Six-to-eight-week-old mice were anesthetized intraperitoneally with pentobarbital at a dose of 50 $mg \cdot kg^{-1}$, and then the LPS (1.25 $mg \cdot kg^{-1}$) or PBS was placed into the trachea using a pipette tip. Mice were sacrificed 12 hours after LPS administration. The middle lobe of the right lung was used for qRT-PCR analysis, and the rest were frozen in the cryotube for Western blot. The left lung was used to collect BALF, and it was then used for histological evaluation.

BALF analysis

BALF (1.0 mL) was obtained through tracheal intubation from lungs, and then centrifuged immediately at 6000 rpm for 10 minutes at 4°C. The BALF supernatant was immediately stored at -80°C to examine the cytokine levels and BALF total protein. The sediment was resuspended in 1 mL PBS and then the total number of cells was counted. The remaining cells were subjected to Wright-Giemsa staining for classification count. The number of neutrophils in 200 cells was counted and classified using a microscope. Total protein levels in BALF were measured using a Pierce™ BCA protein assay kit (#23225, Thermo Fisher Scientific) according to the manufacturer's instructions.

Histological assessment

Lungs were internally fixed by injecting 0.4 mL 4% paraformaldehyde, and then removed into 4% paraformaldehyde for external fixation. Sections (5 µm) were sliced and stained with hematoxylin and eosin (H & E) to demonstrate general morphology. Subsequently, they were visualized with an Olympus BX53 inverted microscope (Olympus, Melville, NY, USA). The lung tissue inflammation score was graded semi-quantitatively, according to published guidelines, as follows: 0, normal; 1, few cells; 2, a ring of inflammatory cells 1 cell layer deep; 3, a ring of inflammatory cells 2-4 cells deep; 4, a ring of inflammatory cells of >4 cells deep. At least five fields were analyzed randomly in each section. All slides were examined in a double-blind manner.

ELISA assay

BALF supernatants were centrifuged at 400 ×*g* for 10 minutes. The inflammatory protein CXCL2 in the supernatants was determined using ELISA kits from MultiScience Biotech following manufacturer's instructions.

LPS-induced peritonitis

Six-to-eight-week-old mice were intraperitoneally injected with LPS (2mg kg^{-1}). After 24 hours, mice were euthanized and peritoneal cells were collected by peritoneal lavage. Then the total cells were counted and then the neutrophils were classified as BALF analysis.

MitoSOX assay

The cells were treated with LPS for 6 hours, and then incubated with 5 µM of mitochondrial superoxide indicator MitoSOX at 37°C for 10 min. At the end of incubation period, dissociated

the cells from the petri dish. Flow cytometric analysis were performed using the phycoerythrin (PE) channel. Data were quantified as medium fluorescent intensity (MFI).

Transmission electron microscopy

After six-hour LPS stimulation, cultured cells were harvested and fixed in 2.5% glutaradehyde overnight at 4˚C, and subsequently post-fixed in 1% osmium tetroxide for 1 hours on room temperature, dehydrated in gradient series of ethanol (70-100%), immersed in propylene oxide as a transition medium, and embedded in pure epoxy resin and polymerized for 48h at 60°C. Then, 90-nm thick sections were prepared using an ultramicrotome, stained with 2% aqueous uranyl acetate and Reynold's lead citrate. Finally, the images were observed using transmission electron microscopy (Tecnai G2 Spirit 120keV) at 65,000× magnification by a blinded observer. The mitochondrial morphology of primary macrophages was divided into two categories. Normal cells presented an intact network of tubular mitochondria, fragmented cells displayed predominantly spherical mitochondria and the loss of their cristae and double-layer membrane in mitochondria.

Structured illumination microscopy

Structured illumination microscopy (SIM) super-resolution images were taken on a Nikon N-SIM system with an oil immersion objective lens 100×, 1.49 NA, Nikon. Images were captured using Nikon NIS Elements and reconstructed using slice reconstruction in NIS elements. Images of fixed cells for N-SIM were taken using z-stacks with step sizes of 0.2 μm.

Statistical analysis

Data are presented as the mean ± standard error of the mean (SEM) of at least three independent experiments. The statistical significance between two groups was calculated using a two-tailed unpaired Student’s *t*-test, and multiple-comparison testing was performed using a one-way ANOVA followed by Bonferroni’s correction. For all tests, statistical significance was recognized when $p \leq 0.05$. Statistical analysis was performed using the GraphPad Prism.

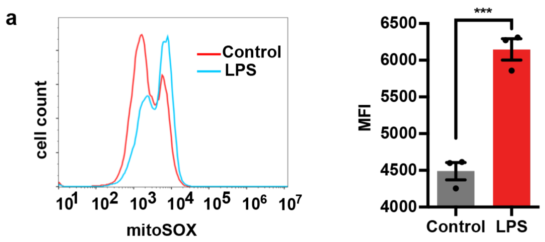

Figure. S1.

LPS induced the production of mitochondrial ROS. a The cells were treated with LPS for 6h and then the mitochondrial ROS was measured using mitoSOX assay (n=3). Data were quantified as medium fluorescent intensity (MFI). Data are presented as means ± SEMs. ***p < 0.001.

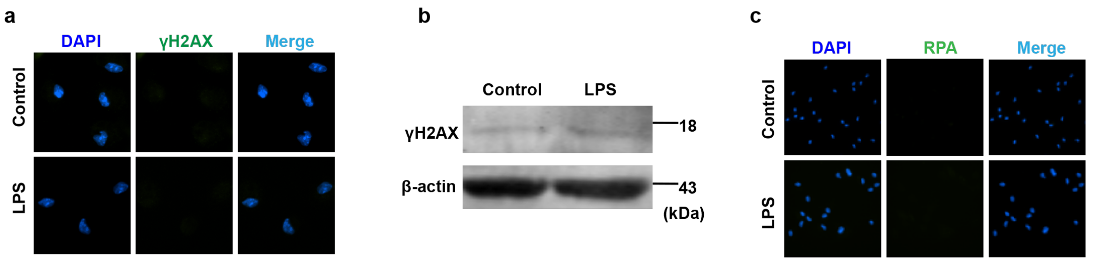

Figure. S2.
DNA damage response in LPS-stimulated primary macrophages. a, b Fluorescent microscopy and western blot for the expression of γH2AX in primary macrophages with LPS stimulation for 6 hours. **c** Primary macrophages were treated with LPS for 24 hours, and representative images of RPA were showed using immunofluorescence. All experiments were repeated at least three times.

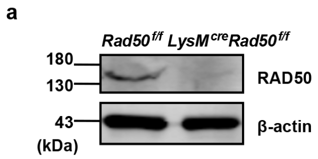

Figure. S3.
The knockout efficiency of RAD50. a Western blot was performed for RAD50 knockout efficiency using primary macrophages from *LysM*^{cre}*Rad50*$^{flox/flox}$ and *Rad50*$^{flox/flox}$ mice.

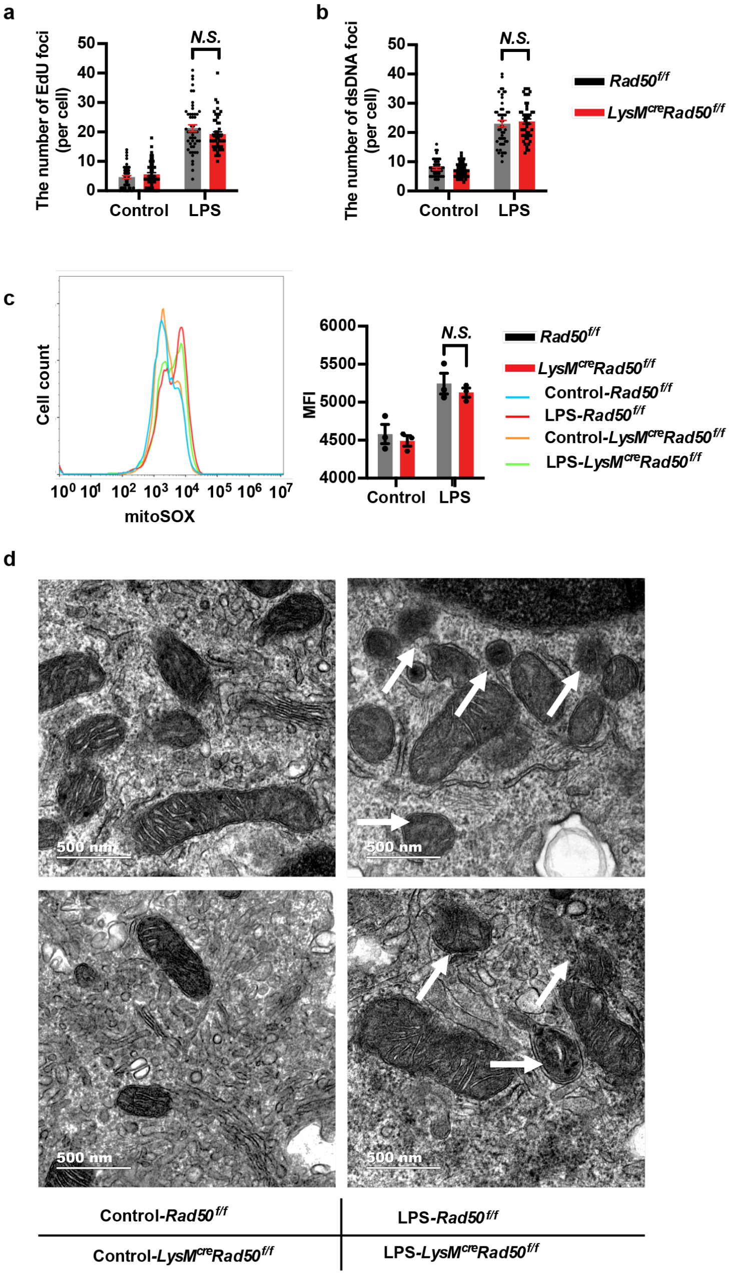

Figure. S4.

RAD50 deficiency exerted no significant protection on LPS-induced mitochondrial injury in macrophages. Primary macrophages from *LysM^{cre}Rad50$^{flox/flox}$* and *Rad50$^{flox/flox}$* mice were incubated with or without EdU and LPS for 6 hours, **a** the number of EdU foci in each cell was analyzed using immunofluorescence, 50 cells per group at least. (Nikon, Eclipse Ti2, original magnification, ×400) **b** The extranuclear dsDNA foci was counted for each macrophage, 50 cells per group at least. (original magnification, ×400) **c** The mitochondrial ROS was measured using a mitoSOX-based assay and accessed by MFI using FACS analysis (n=3). **d** Images of the morphological structures of mitochondria in macrophages were observed by a transmission electron microscopy (TAM) under ×65000 magnification. Cells under LPS exposure displayed

predominantly spherical mitochondria and the loss of their cristae and double-layer membrane (white arrows). Shown are representative images of mitochondria in each group. Scale bars, 500 nm. Results are means ± SEMs. N.S. $p>0.05$.

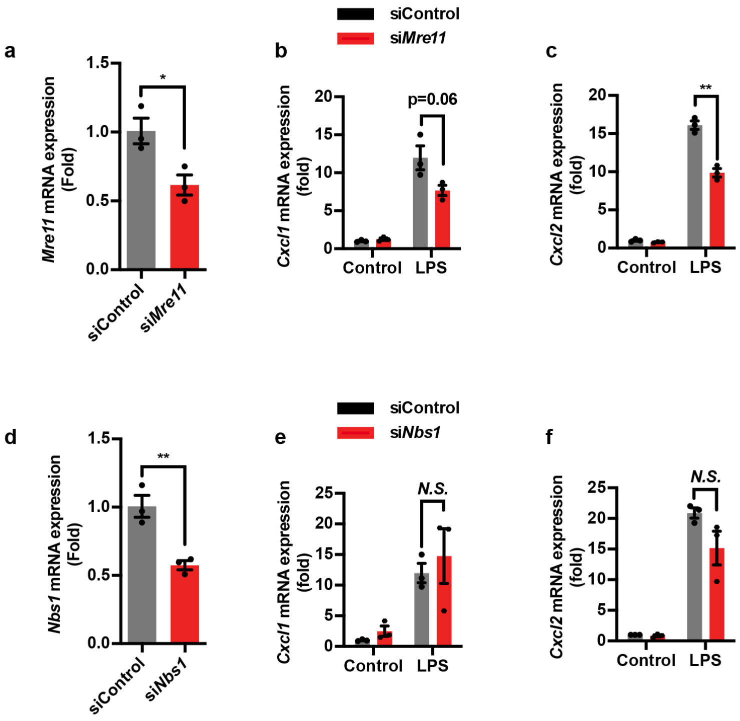

Figure. S5.

The function of MRE11 and NBS1 in LPS-induced macrophage inflammation. a-f Primary macrophages from WT mice were transfected with *Mre11* or *Nbs1* siRNA for 24 hours, then stimulated with LPS for 6 hours (n=3). Relative mRNA levels of *Mre11, Nbs1, Cxcl1 and Cxcl2* were determined using quantitative PCR. Data are presented as means ± SEMs. *p < 0.05, **p < 0.01, *N.S.* p>0.05.

a

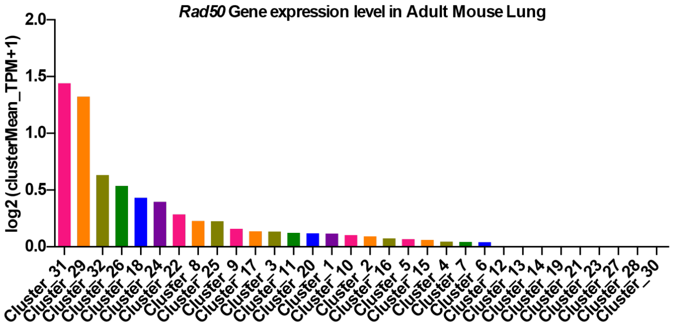

b

Cluster	Annotation
Cluster_31	Myeloid cell_Elane high
Cluster_29	conventional dendritic cell_H2-M2 high
Cluster_32	conventional dendritic cell_Tubb5 high
Cluster_26	Alveolar macrophage_Pclaf high
Cluster_18	Ciliated cell
Cluster_24	CD8+ T cell_Hmgb2 high
Cluster_22	Dividing dendritic cells
Cluster_8	Interstitial macrophage

Figure. S6.

Analysis of relative *Rad50* gene expression in adult mouse lung tissue based on mapping data[3].

a The mapping data was obtained from Mouse cell Atlas, and relative gene expression level of *Rad50* was analyzed. **b** The top 8 cell populations that highly express *Rad50*.

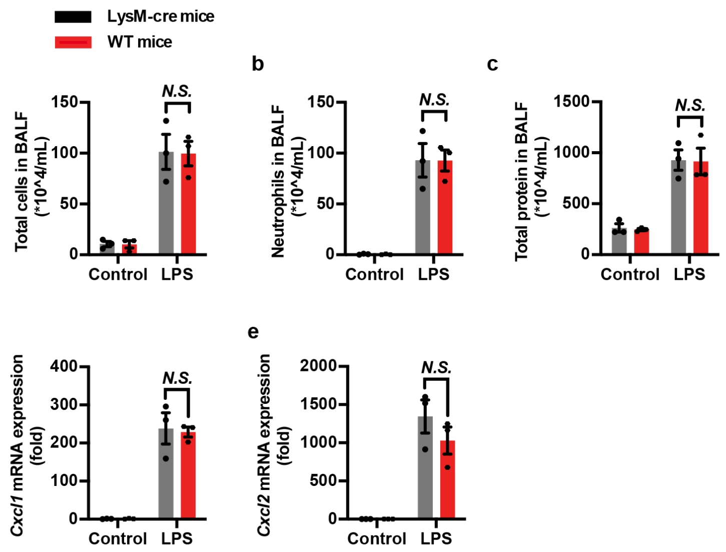

Figure. S7.

The LysM-cre gene editing in C57/BL6 mice exerts no effects on LPS-induced acute lung injury. LysM-cre mice and WT mice (n=3 per group) were injected intraperitoneally with LPS (1.25mg·kg^{-1}) or PBS for 12 hours. **a, b** The total inflammatory cells and neutrophils in BALF were quantified. **c** Total protein in BALF was examined by a BCA assay. **d, e** Relative mRNA levels of *Cxcl1 and Cxcl2* were examined using quantitative PCR. Data are presented as means ± SEMs. *N.S.* p>0.05.

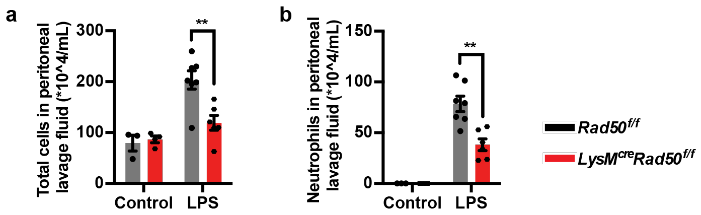

Figure. S8.

Myeloid *Rad50* deficiency attenuated inflammatory responses in LPS-induced peritonitis. *Rad50*$^{flox/flox}$ and *LysM*^{cre}*Rad50*$^{flox/flox}$ mice (n=3-7 per group) were injected intraperitoneally with LPS (2mg·kg^{-1}) or the equivalent volume of PBS as control for 24 hours. In the peritoneal lavage fluid, **a** the total number of inflammatory cells was quantified, **b** the number of neutrophils was calculated. Data are presented as means ± SEMs. **p < 0.01.

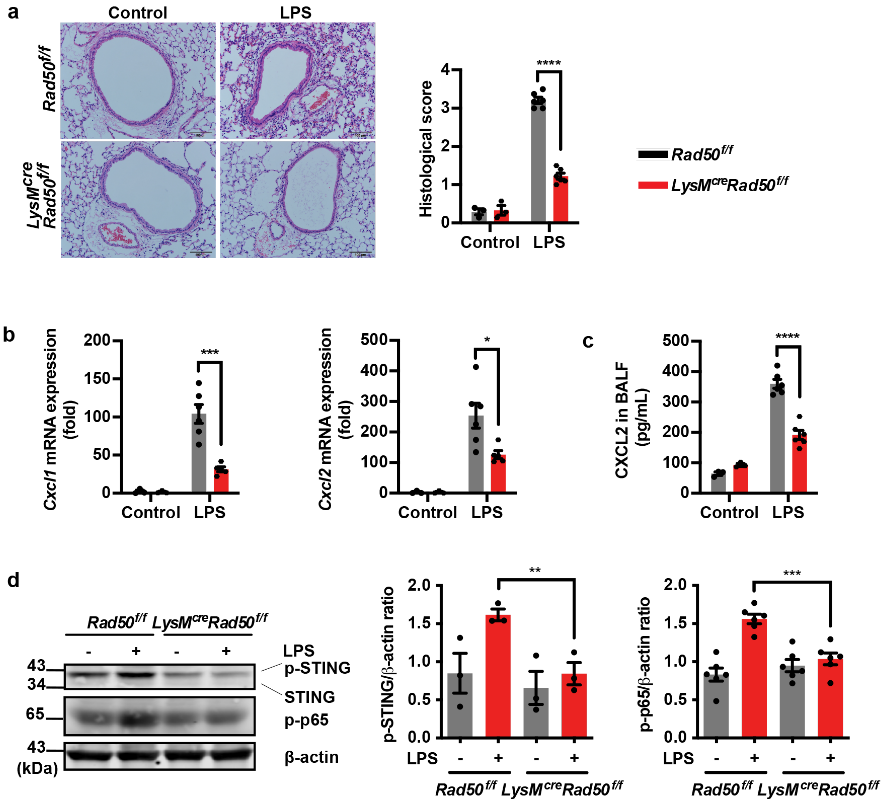

Figure. S9.

Myeloid *Rad50* deficiency attenuated inflammatory responses in LPS-induced acute lung injury. *Rad50*$^{flox/flox}$ and *LysM*^{cre}*Rad50*$^{flox/flox}$ mice (n=3-6 per group) were instilled intratracheally with LPS (1.25 mg·kg^{-1}) or the equivalent volume of PBS as control for 12 hours. **a** Representative images of H & E staining of lung tissues are showed and semi-quantified inflammation score of the H & E staining, five fields at least were examined in each section. **b** *Cxcl1* and *Cxcl2* expression in the LPS-stimulated lung tissues were determined using quantitative PCR. **c** CXCL2 expression in BALF was examined by a quantified ELISA kit. **d** p-STING and p-p65 expression were examined by western blot. Data are presented as means ± SEMs. *p <0.05, **p < 0.01, ***p < 0.001, ****p < 0.0001.

References

1. Clausen, B. E., Burkhardt, C., Reith, W., Renkawitz, R. & Förster, I., Conditional gene targeting in macrophages and granulocytes using LysMcre mice. *Transgenic Res.* **8**, 265-277 (1999).
2. Adelman, C. A., De S & Petrini, J. H., Rad50 is dispensable for the maintenance and viability of postmitotic tissues. *Mol. Cell. Biol.* **29**, 483-492 (2009).
3. Han, X. *et al.*, Mapping the Mouse Cell Atlas by Microwell-Seq. *Cell* **172**, 1091-1107 (2018).
